# Surveillance of laboratory exposures to human pathogens and toxins, Canada, 2024

**DOI:** 10.14745/ccdr.v51i101112a04

**Published:** 2025-12-12

**Authors:** Emily F Tran, Audrey Gauthier, Antoinette N Davis, Christine Abalos, Samuel Bonti-Ankomah

**Affiliations:** 1 Regulatory, Operations and Emergency Management Branch, Public Health Agency of Canada, Ottawa, ON

**Keywords:** Centre for Biosecurity, human pathogens and toxins, laboratory-acquired infections, laboratory exposures, laboratory incidents, Laboratory Incident Notification Canada, surveillance

## Abstract

**Background:**

Exposure incidents to human pathogens and toxins (HPTs) in licensed facilities in Canada are monitored by Laboratory Incident Notification Canada (LINC), a surveillance system that describes and identifies trends among exposure incidents in Canada using quantitative and qualitative data.

**Methods:**

Confirmed exposure incidents reported to LINC in 2024 were analyzed. The exposure incident rate was calculated and compared to previous years. A seasonality analysis compared monthly trends. Exposure incidents were described by sector, implicated HPTs, main activity, occurrence types, root causes, affected individuals and reporting delay. Text-based descriptions of exposure incidents underwent qualitative analysis.

**Results:**

In 2024, there were 71 confirmed exposure incidents affecting 132 individuals. There were 67.5 incidents per 1,000 active licences. Bacteria was the most commonly implicated HPT (64%). Microbiology (67.6%) was the primary activity during confirmed exposures. The public health sector had the highest incident rate and mean number of affected persons per active licence. The most frequently reported occurrence type and root cause was procedure-related (21.4%) and human factors (62%), respectively. Most affected individuals were technicians/technologists (76.5%). The median time between incident and reporting was five days.

**Conclusion:**

The exposure incident rate was higher in 2024 compared to the previous year. The public health sector had the highest incident rate between 2016–2024. Qualitative analysis revealed that working with cultures outside the biological safety cabinet and insufficient face-related personal protective equipment were common factors involved in confirmed exposure incidents.

## Introduction

Research involving human pathogens and toxins (HPTs), such as bacteria, viruses and fungi, can provide valuable insight into developing protective measures against harmful biological agents. However, work involving HPTs carries numerous risks, including potential exposure incidents that may lead to laboratory-acquired infections (LAIs) or intoxications ((1,2)). In rare cases, these incidents can extend beyond the facility and affect public health ((2,3)).

In Canada, more than 1,000 licensed facilities conduct activities involving HPTs. These facilities are regulated by the Public Health Agency of Canada (PHAC) under the *Human Pathogens and Toxins Act* (HPTA) ((4)) and its associated *Human Pathogens and Toxins Regulations* (HPTR) ((5)), which mandate the reporting of exposure incidents involving Risk Group (RG) 2 or higher pathogens. The RG classification is assigned based on inherent characteristics and describes the risk of a pathogen at the individual-level and population-level. To monitor exposures to RG2, RG3 and RG4 HPTs in licensed facilities throughout Canada and support licensed facilities with mitigation of recurrence, a federal surveillance system, Laboratory Incident Notification Canada (LINC), was launched by PHAC in December 2015.

Since its inception, LINC has published annual reports summarizing exposure incidents in Canada and provided data on a number of variables associated with these exposure incidents ((6–13)). These reports have often found that confirmed exposures occurred particularly during microbiology or *in vivo* animal research activities ((9–13)). Common occurrence types involved issues with procedures, sharps and personal protective equipment (PPE) ((6–13)). Frequent root causes are issues with standard operating procedures (SOPs) and human factors-related issues ((6–13)). Most affected individuals worked as laboratory technicians or technologists ((8–13)). The most commonly implicated biological agents are RG2 bacteria and viruses ((6–11,13)).

Canada remains one of the few countries with a dedicated surveillance system focused on exposures to biological agents. Some countries (Australia, the United States and Singapore) have their own surveillance programs that focus on specific risk groups or elements of biosafety ((14–17)). In Australia, Security Sensitive Biological Agents (SSBAs), a subset of pathogens posing higher biosecurity risks due to their potential to be used for biological weapons, are monitored ((14,18)). In the United States, the possession, use and transfer of biological select agents and toxins are monitored under the Federal Select Agent Program (FSAP) ((15)). In Singapore, infections, illnesses, adverse events and incidents involving certain agents or toxins must be reported to the Ministry of Health ((16,17)). Comparative analyses between LINC’s incident reports and those from other countries remain limited due to differences in data collection and availability, particularly regarding exposure incident characteristics. However, LAIs reported in the literature from other countries align with LINC’s data. Globally, procedural errors and sharps-related incidents are common sources of LAIs ((1,2,19–22)), with most incidents occurring during microbiology activities ((1,2,20)) and laboratory technicians as the most frequently affected personnel in these incidents ((1,20)).

With the overall goal of enhancing biosafety awareness and improving laboratory practices in Canada, LINC’s annual reports have provided insights to help minimize exposure incidents. The objective of this report is to analyze exposure incidents reported to LINC in 2024. These findings will further enhance the understanding of factors contributing to exposure incidents and inform targeted areas for biosafety training and best practices.

## Methods

### Data source and collection

Under the HPTA and HPTR, in the event of an HPT-related incident, all licensed Canadian facilities must report specific details to PHAC without delay. The Agency’s web-based Biosecurity Portal, which uses Microsoft Dynamics’ Customer Relationship Management (CRM) software, provides standardized forms for licensed facilities to report laboratory exposures, non-exposures and other incidents. These forms contain data validation rules to prevent missing mandatory data. Each report is monitored and processed by LINC in the Integrated Suite of Tools for Operational Processes (iSTOP) and undergoes manual verification by LINC to ensure completeness. A biocontainment review is conducted following receipt of incident reports. For each exposure report, a subsequent follow-up report providing updates and additional incident investigation details, such as root causes and corrective actions, is required and analyzed. If a follow-up report was not submitted, information from the initial exposure report is used.

Incident data from January 1, 2024, to December 31, 2024, were collected through the Biosecurity Portal and extracted from iSTOP on January 10, 2025, and analyzed for this article. The total number of active licences per sector was extracted from iSTOP on January 29, 2025. The analysis included reports that did not have a specific incident date, in which case the submission date was used.

### Report variables

Several variables were used to describe the confirmed exposure incident reports in 2024, including exposure report type, activity being performed during the incident, occurrence types, root causes, reporting delay, licence sector, number of active licences, type of HPTs involved, and description of the incident. Definitions for the main activities and occurrence types are provided in the **Appendix, ****Table A1**** and ****Table A2**. Information on affected individuals, such as their role, highest level of education and years of laboratory experience, was also collected. Other demographic data, such as gender, age and income, are not collected by LINC.

### Analysis

The analysis was performed in R 4.2.2. Plots and tables were generated in R and Microsoft Excel, which was also used for data validation. This study also reviewed data from 2016–2023 to incorporate any updates to previously submitted reports.

### Quantitative data analysis

Follow-up reports were reviewed to classify exposure incidents reported to LINC in 2024 as either confirmed exposures, including LAIs, or ruled out. Ruled out exposure incidents, which were excluded from the main analysis, include those ruled out after investigation, exposures involving RG1 pathogens (which are unregulated), and exposures that are not mandated under the HPTA and HPTR, such as incidents involving primary specimens or unlicensed laboratories. The incident rate of exposures to HPTs was calculated by dividing the number of reported exposure incidents by the total number of active licences during the surveillance period. The 2024 exposure incident rate was compared to rates from previous years and the baseline incident rate from 2016 to 2023. The baseline incident rate was calculated by dividing the total number of exposure incidents between 2016–2023 by the total number of active licences between 2016–2023. Descriptive statistics were obtained for continuous and categorical quantitative variables. Wilcoxon signed rank tests were used to compare exposure incident rates per sector in 2024 with those of 2016–2023. The t-tests were used to compare the monthly incident rates in 2024 with those of 2016–2023.

### Qualitative data analysis

The text-based variables extracted from iSTOP followed an unstructured format, similar to journal entries. To transform the text into structured data, the variables underwent text preprocessing, including the removal of stop words, punctuation and whitespace. Through tokenization, the text was broken down into individual words, which were then analyzed for frequency and visualized in a plot. A manual revision of the text-based variables was also conducted for cross-validation.

## Results

**Figure 1:**
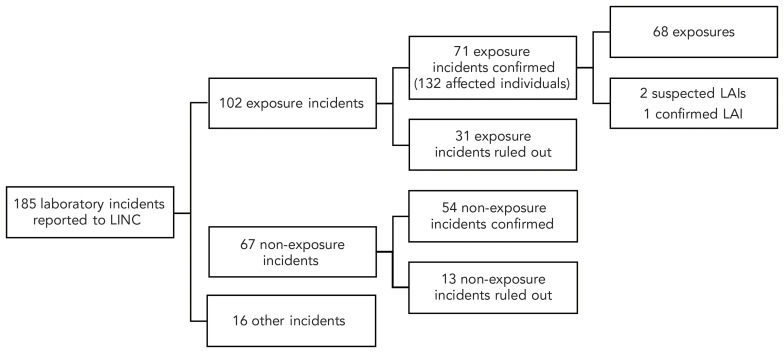
Incidents reported to Laboratory Incident Notification Canada, 2024 Abbreviations: LAIs, laboratory-acquired infections; LINC, Laboratory Incident Notification Canada

Between January 1, 2024 and December 31, 2024, LINC received 185 laboratory incident reports (**Figure 1**). Of these, 102 (55.1%) were exposure incidents, 67 (36.2%) were non-exposure incidents, and 16 (8.6%) were other incidents. The exposure incidents included 71 confirmed exposure incidents and 31 ruled out exposure incidents. The 71 confirmed exposure incidents, which involved 132 affected individuals, were further categorized into 68 (95.8%) confirmed exposures, two (2.8%) suspected LAIs, and one (1.4%) confirmed LAI.

### Annual and monthly exposure incident rate trends

**Figure 2:**
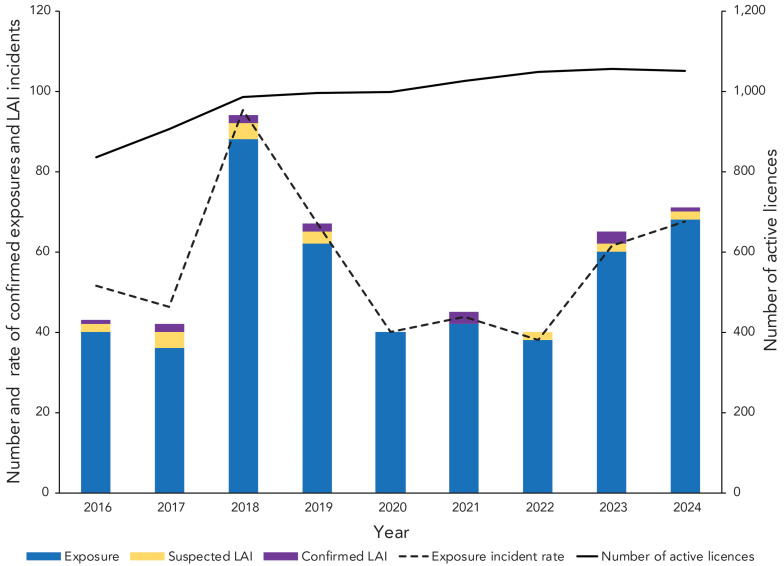
Confirmed exposure incidents, suspected and confirmed laboratory-acquired infections, active licences and exposure incident rate, 2016–2024 Abbreviation: LAI, laboratory-acquired infection

In 2024, there were 1,052 active licences, of which 975 (92.7%) were for RG2 HPTs, 71 (6.7%) for RG3 HPTs, two (0.2%) for RG4 HPTs, and four (0.4%) for SSBAs (data not shown). The exposure incident rate, calculated as the number of confirmed exposure incidents per 1,000 active licences, was 67.5 in 2024. This represented a slight increase from 61.5 in 2023 (**Figure 2**). The annual baseline incident rate from 2016 to 2023 was 55.5 confirmed exposure incidents per 1,000 active licences (95% confidence interval [CI]: 43.2–67.9), an increase from the 2016 to 2022 baseline of 54.6 in the 2023 surveillance report ((13)). Although the 2024 exposure incident rate was higher than the annual baseline incident rate, the difference was not statistically significant (*p*=0.099).

**Figure 3:**
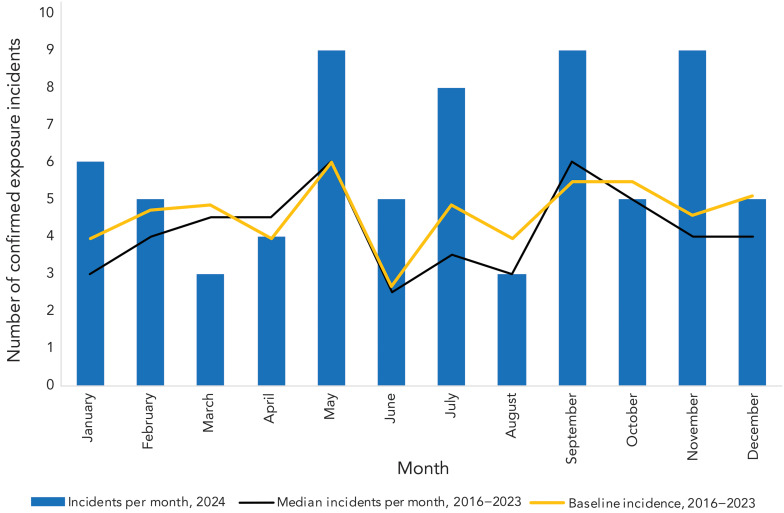
Seasonality analysis using median confirmed exposure incidents per month and baseline incidence, 2016–2024

An average of 5.9 (95% CI: 4.7–7.1) confirmed exposure incidents per month was observed in 2024, which did not differ significantly from the monthly average of 4.5 (95% CI: 4.0–5.1) from 2016 to 2023 (*p*=0.07). The seasonality trend differed slightly in 2024 compared to the 2016–2023 median incidents per month and the baseline incidence, with the number of confirmed exposure incidents in 2024 peaking in May, September and November (n=9; 12.7% each) (**Figure 3**). Compared to the 2016–2023 median number of incidents per month, the frequency of incidents in 2024 was lower than expected in March (n=3; 4.2%) and nearly doubled in January (n=6; 8.5%), June (n=5; 7.0%), July (n=8; 11.3%) and November (n=9; 12.7%).

### Implicated human pathogens and toxins

Of the 75 known implicated HPTs, 64% (n=48) were RG2 pathogens and 36% (n=27) were RG3 pathogens (**Table 1**). There was one unknown pathogen, not included in Table 1. All of the RG2 pathogens implicated were non-SSBAs, with bacteria being the most frequently reported (n=34; 45.3%). In contrast, only 12% of implicated RG3 pathogens were non-SSBAs, with viruses being most commonly reported (n=4; 5.3%). All implicated SSBAs were RG3 pathogens and accounted for 24% of all HPTs, with bacteria being the most frequently involved pathogen (n=13; 17.3%).

**Table 1:** Known human pathogens and toxins implicated in reported exposure incidents by risk group level and the biological agent’s security sensitive status, 2024 (n=75)

Biological agent type by risk group	Non-SSBA		SSBA		Total	
	n	%	n	%	n	%
**RG2**	**48**	**64.0**	**0**	**0**	**48**	**64.0**
Bacteria	34	45.3	0	0	34	45.3
Fungus	3	4.0	0	0	3	4.0
Parasite	0	0	0	0	0	0
Prion	2	2.7	0	0	2	2.7
Toxin	3	4.0	0	0	3	4.0
Virus	6	8.0	0	0	6	8.0
Cell line	0	0.0	0	0	0	0
**RG3**	**9**	**12.0**	**18**	**24.0**	**27**	**36.0**
Bacteria	1	1.3	13	17.3	14	18.7
Fungus	3	4.0	2	2.7	5	6.7
Parasite	0	0	0	0	0	0
Prion	1	1.3	0	0	1	1.3
Toxin	0	0	0	0	0	0
Virus	4	5.3	3	4.0	7	9.3
Cell line	0	0	0	0	0	0
Total	57	76.0	18	24.0	75	100

Abbreviations: RG2, Risk Group 2; RG3, Risk Group 3; SSBA, Security Sensitive Biological Agent

Overall, bacteria and viruses were the leading HPTs implicated in exposure incidents involving both RG2 and RG3 pathogens. Except for parasites and cell lines, which were not implicated in any exposures, prions were the least commonly involved pathogens (2.7% for RG2; 1.3% for RG3). The confirmed LAI in 2024 was associated with *Staphylococcus aureus*, while the two suspected LAIs were linked to *Mycobacterium tuberculosis* and *S. aureus*. The top three most implicated HPTs in 2024 were *Neisseria meningitidis* (n=8; 10.7%), *Brucella melitensis* (n=6; 8.0%), and *S. aureus* (n=6; 8.0%).

### Main activity

Reporters could select from 11 possible main activity types performed during the exposure incident: animal care, autopsy/necropsy, cell culture, education/training, *in vivo* animal research, maintenance, microbiology, microscopy, molecular investigations, serology or “other” (Appendix, Table A1). Microbiology and *in vivo* animal research were the most commonly reported activities in 2024, accounting for 67.6% and 8.5% of confirmed exposure incidents, respectively. The remaining activities were less frequently reported, each accounting for 1.4% to 5.6% of confirmed exposure incidents (data not shown).

### Sector

Licensed facilities could belong to one of eight sectors: academic, do-it-yourself (DIY) biology, environmental health – government (environmental health), hospital, other government, public health – government (public health), private industry/business, or veterinary/animal health – government (veterinary/animal health). The other government sector includes facilities that handle HPTs at the federal, provincial/territorial and municipal levels, but are not classified under environmental health, public health or veterinary/animal health. The DIY biology sector, whose data was first analyzed and presented in 2024 ((13)), includes any individual conducting their own experiments who is not working within an institutionalized facility. Data for the “not specified” sector was also first analyzed in 2024 ((13)).

**Figure 4:**
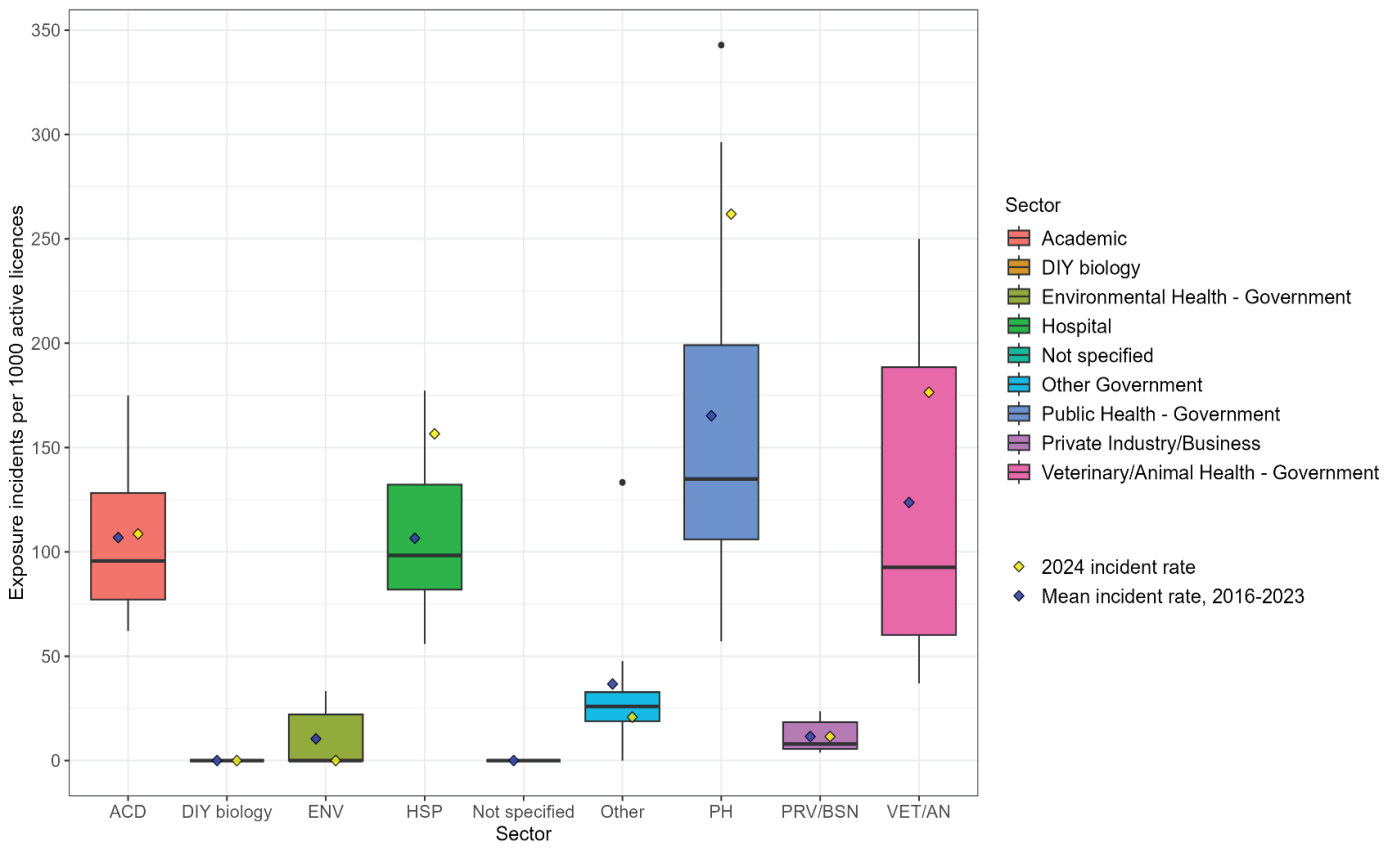
Exposure incidents per 1,000 active licences (exposure incident rates) by sector, 2016–2024^a^ Abbreviations: ACD, academic; DIY biology, do-it-yourself biology; ENV, environmental health – government; HSP, hospital; Other, other government; PH, public health – government; PRV/BSN, private industry/business; VET/AN, veterinary/animal health – government ^a^ The boxplots summarize the exposure incidents per 1,000 active licences between 2016 and 2023. For each sector, the yellow diamond corresponds to the 2024 exposure incident rate, and the blue diamond corresponds to the mean exposure incident rate from 2016 to 2023. The black dot corresponds to an outlier. There is no yellow diamond for the incidents with no specified sector because there were no licences associated with these cases in 2024; the exposure incident rate for the cases with no specified sector reflects data from 2023 only

Between 2016 and 2023, the mean and median exposure incident rates were highest in the public health sector (mean=165.3, median=135.0), followed by veterinary/animal health (mean=123.7, median=92.6), academic (mean=106.9, median=95.7), and hospital sectors (mean=106.5, median=98.3) (**Figure 4**). Excluding the DIY biology sector and cases where the sector was not specified, the environmental health sector (mean=10.5, median=0) and private industry/business sector (mean=11.5, median=7.9) had the lowest incidence rates. The greatest variation in exposure incident rates was observed in the veterinary/animal health sector (interquartile range [IQR]: 128.3), while the private industry/business sector had the least variation (IQR: 12.9), excluding the DIY biology sector.

The 2024 exposure incident rate patterns mirrored trends observed between 2016 and 2023; however, incident rates in the hospital (incidence=156.6) and public health (incidence=261.9) sectors were statistically significantly higher (*p*<0.05) (Figure 4) in 2024 than trends from previous years. While exposure incident rates in the environmental health (incidence=0) and other government (incidence=20.8) sectors were visibly lower than those between 2016 and 2023, the differences were not statistically significant (*p*>0.35). Among the LAIs reported in 2024, one confirmed and one suspected case occurred in the hospital sector, while one suspected LAI occurred in the academic sector.

### Occurrence types

**Figure 5:**
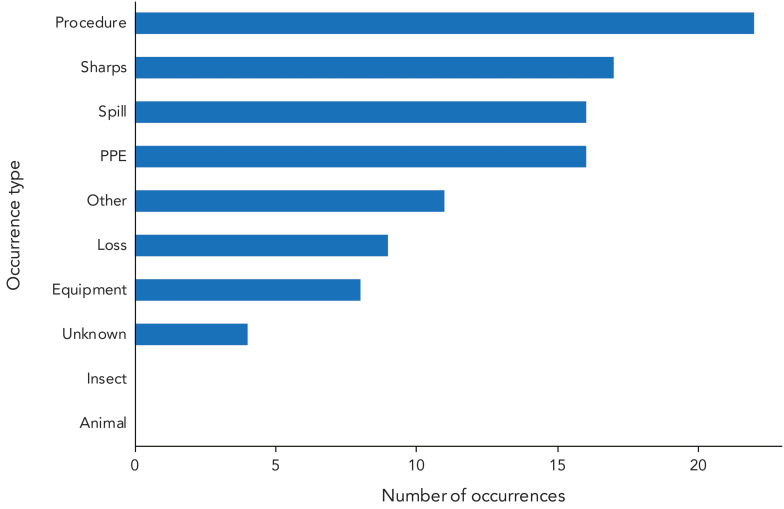
Occurrence types involved in confirmed exposure incidents, 2024 (n=103) Abbreviation: PPE, personal protective equipment

Confirmed exposure incidents could have one or more of ten occurrence types (Appendix, Table A2). There were 103 occurrence types cited in 2024. Procedure-related occurrences (n=22; 21.4%) were the most common, followed by sharps (n=17; 16.5%), spill (n=16; 15.5%) and PPE (n=16; 15.5%) (**Figure 5**). The least cited occurrence types were unknown (n=4; 3.9%) and animal and insect occurrences, which were not cited in any confirmed exposure incidents.

### Root causes

There are six main root causes that can be associated with an exposure incident: human factors, SOPs, training, management/oversight, equipment, and communication. Examples of areas of concern for each of these categories are presented in **Table 2**. Most confirmed exposure incidents cited multiple root causes (n=167), averaging 2.35 root causes per confirmed exposure (Table 2). Human factors were the most common root cause (n=44; 62%), followed by SOP (n=27, 38%).

**Table 2:** Root causes reported in follow-up reports of confirmed exposure incidents, 2024 (n=167)

Root cause	Examples of areas of concern	Citations	
		n	%^a^
Human factors	A violation (cutting a corner, not following correct procedure, deviating from SOP)	44	62.0
	An error (a mistake, lapse of concentration, or slip of any kind)		
SOP	Documents were followed as written but not correct for activity/task	27	38.0
	Procedures that should have been in place were not in place		
	Documents were not followed correctly		
Training	Training was not in place but should have been in place	23	32.4
	Training was not appropriate for the task/activity		
	Staff were not qualified or proficient in performing task		
Management and oversight	Supervision needed improvement	20	28.2
	Lack of auditing of standards, policies and procedures		
	Risk assessment needed improvement		
Equipment	Equipment quality control needed improvement	24	33.8
	Equipment failed		
	Equipment was not appropriate for purpose		
Communication	Communication did not occur but should have	18	25.4
	Communication was unclear, ambiguous, etc.		
Other	Root causes not captured elsewhere	11	15.5
	Unpredictable/random movement by research animal		

Abbreviation: SOP, standard operating procedure

^a^ Percentage of exposure incidents associated with this root cause

### Affected individuals

**Figure 6:**
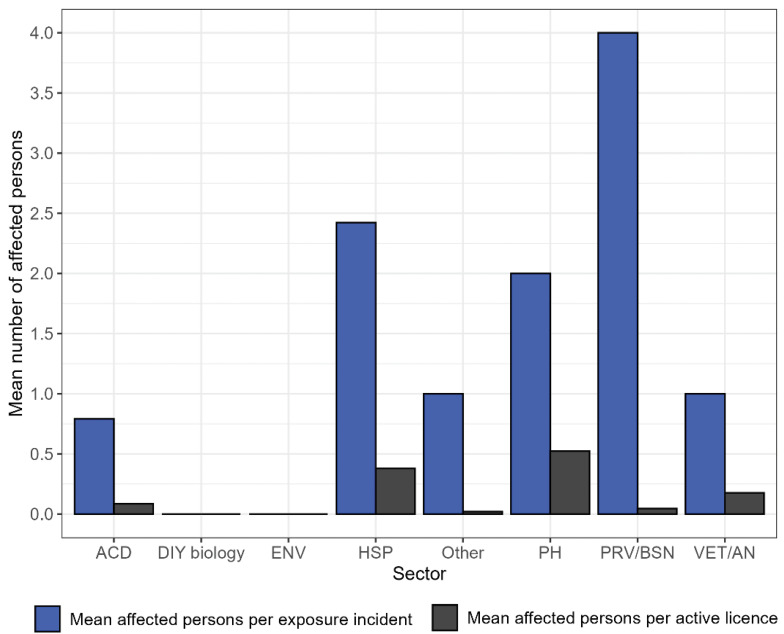
Mean number of affected persons per exposure incident and per active licence by sector, 2024 Abbreviations: ACD, academic; DIY biology, do-it-yourself biology; ENV, environmental health – government; HSP, hospital; Other, other government; PH, public health –government; PRV/BSN, private industry/business; VET/AN, veterinary/animal health – government

A total of 132 individuals were affected in the confirmed exposure incidents, averaging 1.86 affected persons per incident. The three sectors with the highest mean number of affected persons per confirmed exposure incident were private industry/business (mean=4.00), hospital (mean=2.42) and public health (mean=2.00), while the lowest mean was in academic sector (mean=0.79) (**Figure 6**). The public health and hospital sectors also had the highest mean number of affected persons per active licence (mean=0.52 and mean=0.38, respectively), while the other government sector had the lowest (mean=0.02) (Figure 6).

**Figure 7:**
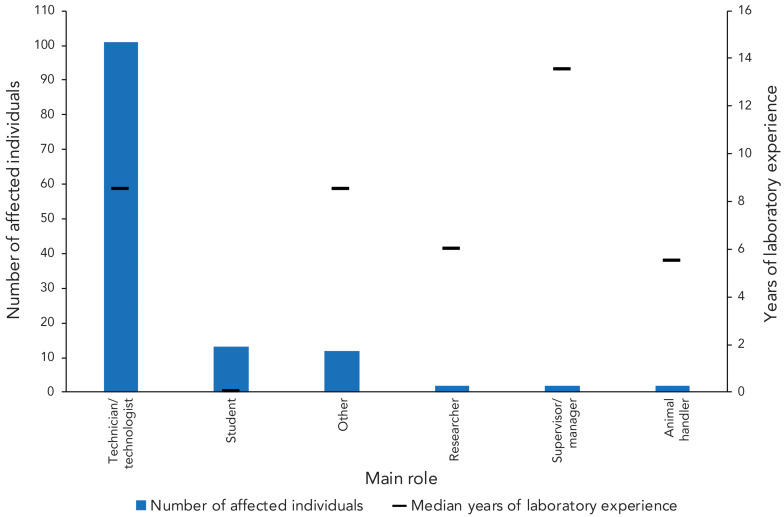
Affected individuals in confirmed exposure incidents reported by number of years of laboratory experience and main role, 2024 (n=132)

The majority of affected individuals were technicians/technologists (n=101; 76.5%) with a median of 8.5 years of laboratory experience (**Figure 7**). Students (n=13; 9.8%) were the second most affected individuals and had the least laboratory experience (median=0 years). Among the roles with the lowest number of affected individuals (n=2; 1.5% each) were supervisors/managers, who had the most laboratory experience (median=13.5 years), researchers (median=6 years), and animal handlers (median=5.5 years).

### Reporting delay

**Figure 8:**
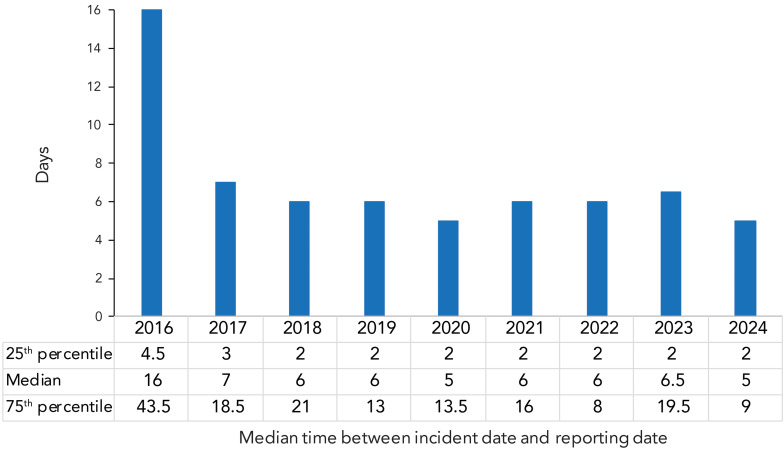
Time between when the confirmed exposure incident happened and when it was reported to Laboratory Incident Notification Canada, 2016–2024

Reporting delay refers to the time between when the exposure incident actually happened and when it was reported to PHAC through LINC. The median time between incident date and reporting date in 2024 was five days, the shortest duration since 2020 (**Figure 8**). Compared to previous years, with the exception of 2022, there was also less variation in the reporting delay in 2024, with an IQR of seven days, approximately 50% less than in most past years.

### Qualitative analysis

**Figure 9:**
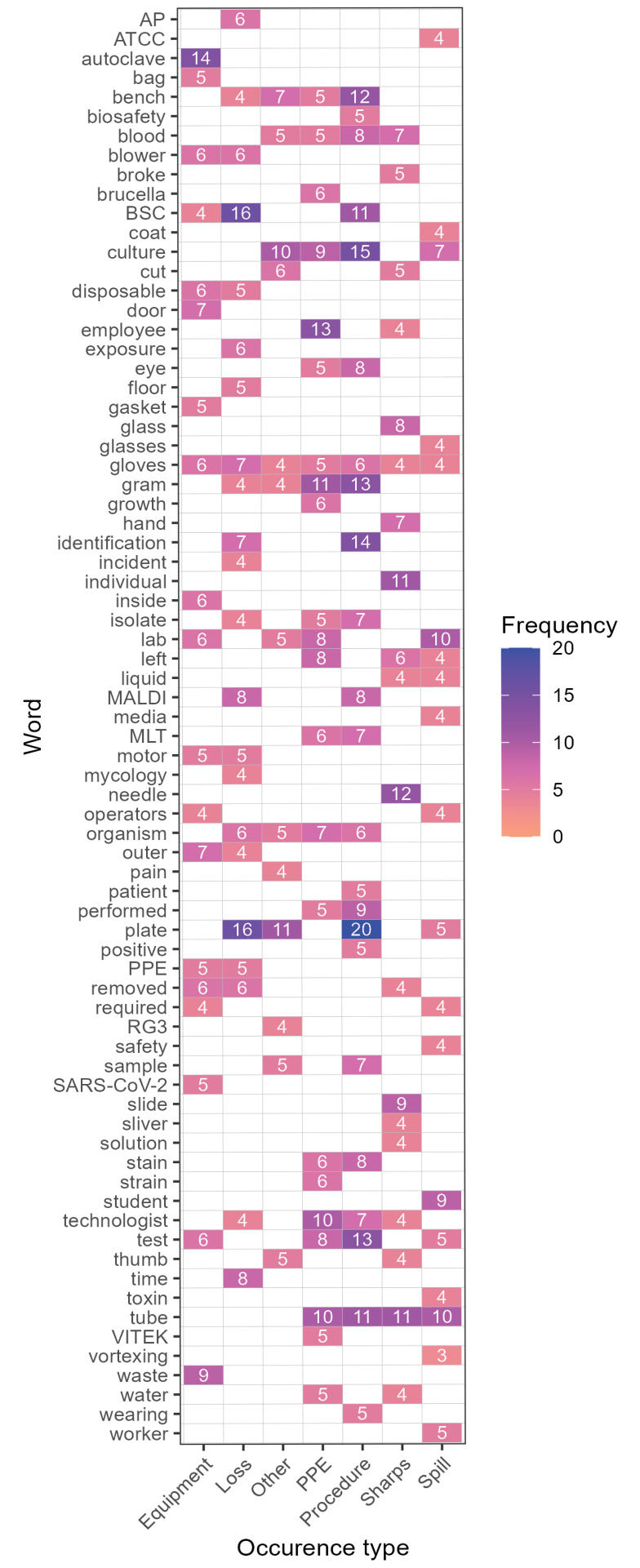
Heatmap of most frequently used words to describe confirmed exposure incidents, 2024^a^ Abbreviations: AP, affected person; ATCC, American Type Culture Collection; BSC, Biological Safety Cabinet; gram, gram stain test/gram smear; MALDI, matrix-assisted laser desorption/ionization; MLT, medical lab technologist; PPE, personal protective equipment; SARS-CoV-2, severe acute respiratory syndrome coronavirus 2; VITEK, VITEK 2 system (BioMérieux) ^a^ Coloured tiles refer to the frequency of the word. Blank tiles do not indicate absence of word usage in the occurrence type, but that it was not used frequently enough to be considered among the most used words

Seventy of the 71 confirmed exposure incidents provided text-based descriptions of the incidents. The frequencies of the most commonly used words to describe exposure incidents by occurrence type are presented in **Figure 9**. Overall, the most frequent words were “plate” (n=52; 5.7%), “biological safety cabinet” (BSC) and “tube” (each n=42; 4.6%), “culture” (n=41; 4.5%), “gloves” (n=36; 4.0%), “gram” (n=32, 3.5%), “lab” (n=29; 3.2%), “bench” (n=28; 3.0%), “test” (n=26; 2.8%), “blood” and “technologist” (each n=25; 2.8%) and “organism” (n=24; 2.6%). All of these words were mentioned in at least four occurrence types.

The word “plate,” primarily referring to petri dishes/culture plates, appeared across four occurrence types: most frequently in procedure, then loss of containment, “other,” and spill. It was used frequently along with “culture,” which also appeared most often in procedure-related occurrences, “BSC,” and “gram” (referring to gram stain tests for bacteria). Manual examination of the descriptions found that exposures often resulted during identification of HPTs in plates or while working in proximity to cultures or colonies in the plates, particularly on an open bench. The word “tube,” mentioned in occurrences related to PPE, procedure, sharps, and spills, was commonly used to describe liquid spills, issues with vortexing and broken glass from tubes breaking. The word “gloves” was the only one to appear across all occurrence types, mostly used to state that they were being worn during the incident.

## Discussion

An increase in the exposure incident rate was observed in 2024, continuing the upward trend since 2022 ((13)). While this rise may be due in part to a return to pre-COVID-19 working conditions, other factors could have also contributed, including increased familiarity with the incident reporting process. For example, over the past few years, PHAC engaged with stakeholders through conferences, webinars ((23,24)), information bulletins ((25)), e-learning courses ((26)) and inspections to enhance awareness of the surveillance program and reinforce appropriate reporting practices. These actions may have contributed to increased reporting and a reduction in reporting delays in 2024.

Consistent with previous years, microbiology and *in vivo* animal research remained the most frequently reported activities being conducted at the time of the confirmed exposure incident ((9–13)). Primary occurrence types remained procedure-related and sharps-related ((9–13)), while human factors and SOPs continued to be the most commonly cited root causes (8–13). Technicians and technologists represented the majority of affected individuals ((8–13)), and non-SSBAs, RG2 and bacteria remain the most implicated HPTs (9–13). For the second consecutive year, the bacteria *B. melitensis,* emerged as a leading SSBA ((12,13)), a trend also observed in the FSAP ((15)). No RG4 HPTs were implicated in exposure incidents in 2024.

### Public health sector had the highest exposure incident rate

Analysis of the data revealed that the public health sector had the highest exposure incident rate in 2024, and the highest mean exposure incident rate from 2016 to 2023. While no studies indicate that public sector facilities have distinct laboratory practices leading to greater risks, the relatively small number of licences in this sector may contribute to an overrepresentation of incidents in the data. The veterinary/animal health sector had the second-highest exposure incident rate, which could be due to the larger number of possible exposure routes when working with animals (i.e., biting, scratching) compared to *in vitro* settings ((27)) and more risks working with animals. These sectors also had larger IQRs, potentially because of variation of research activity from year-to-year and disruption in activity in 2020 and 2021 due to the COVID-19 pandemic ((11,12,28,29)).

### Increase in affected individuals, differences between sectors

A higher average number of affected persons per confirmed exposure incident was noted in 2024 compared to the last few years, with the exception of 2022 ((10–13)). While an average of 1.57 persons were affected per confirmed exposure incident in 2023, this average rose to 1.86 in 2024. This was due to the greater number of confirmed exposure incidents involving more than five individuals compared to the previous year. Differences in affected persons by sector were also observed in 2024. The private industry/business sector, which historically has had fewer confirmed exposure incidents (Figure 4), had the highest mean number of affected individuals per confirmed exposure. This could indicate that a larger number of individuals were working on the task or were nearby during the confirmed exposure incident. The higher number of affected individuals per licence in the public health and hospital sectors could be attributable to contact with cultured biohazardous samples (i.e., blood cultures) ((29)) and higher risk HPTs. The majority of licensed facilities are containment level 2, where hand-to-face contact transmission might be a significant risk to workers ((30)).

### A qualitative analysis provides more insight about exposure incidents

A qualitative analysis of the confirmed exposure incidents revealed occurrence type-specific trends and overall areas to improve. Procedure-related occurrences frequently involved exposures from working with HPT cultures on an open work bench, instead of working in a BSC, without knowing the cultures consisted of higher risk pathogens (e.g., when opening/examining plates, identifying the organism). This situation was also described in loss of containment-related occurrences. This suggests that stronger emphasis on handling cultures in a BSC is needed to prevent aerosol releases or transmission, even if it may not necessarily be the current common practice ((31,32)). For sharps-related occurrences, finger-pricking from needles (e.g., during re-capping, injection, obtaining samples) or cuts by glass shards from broken tubes/slides were the main sources of exposure, while cuts by scalpels/blades were less common. This could be due to the greater number of tasks involving needles. The increased risk of occupational injuries related to needles and glass shards has been documented in other studies ((33–36)). There should, therefore, be increased caution when performing certain high-risk procedures. Equipment-related occurrences most commonly involved the malfunctioning of the BSC blower motor and tubing issues. Although the word “autoclave” was used most frequently for equipment-related occurrences, it was usually to confirm that it had been used for sterilization. However, there was one incident involving exposure to waste that had not been properly autoclaved. Further, while most of the exposure incident descriptions mentioned that gloves and laboratory coats were worn as PPE, face masks and eye protection equipment tended not to be worn as consistently and partially explained the reason for some of the confirmed exposures.

### Limitations

The use of standardized reporting forms during the incident reporting process over the past nine years remains a strength. It has allowed for the collection of consistent data and enhanced the reliability of trend analysis and identification of biosafety challenges.

Lack of facility-specific details (e.g., size of workforce, breakdown of roles, years of experience of each employee, etc.) remains a limitation. Access to such data could enable more detailed analyses using inferential statistics and hypothesis-driven studies to explore potential factors associated with laboratory exposure incidents. Second, there is a relatively small sample size of confirmed exposure incidents each year, potentially leading to higher variability in the results and challenges in detecting trends. The possibility of underreporting remains, though its scale cannot be estimated. Licence holders can only self-identify in one sector, which does not take into consideration the fact that a facility can operate in more than one sector. As the number of active licences each year is recorded by LINC at the beginning of each year, inaccuracies may occur when conducting analyses retrospectively, since the number can vary slightly throughout the year. Finally, a lack of comparable national incident reporting surveillance systems outside Canada remains a limitation that affects our ability to contextualize these findings and trends internationally ((37)).

## Conclusion

The exposure incident rate continued to increase in 2024 and surpassed all pre-pandemic levels, except for 2018. Most of the confirmed exposure incident characteristics in 2024 were consistent with previous years. Sector-specific analyses found that the public health sector had both the highest exposure incident rate between 2016 and 2024, and the highest number of affected persons per active licence between 2016 and 2024. The qualitative analysis of confirmed exposure incident reports identified that not working with cultures in a BSC and a lack of face-related PPE were common factors in confirmed exposure incidents. The insights from this report can inform enhancements to biosafety guidelines and practices, thereby helping to reduce exposure incidents in licensed Canadian facilities.

## Appendix

**Table A1:** Definitions of main activity

Main activity	Definition
Animal care	Activities such as attending to the daily care of animals and providing animals with treatment
Autopsy or necropsy	Post-mortem surgical examinations for purposes such as determining cause of death or to evaluate disease or injury for research or educational purposes
Cell culture	The process of growing cells under controlled conditions—it can also involve the removal of cells from an animal or plant
Education or training	Education or training of students and/or personnel on laboratory techniques and procedures
*In vivo* animal research	Experimentation with live, non-human animals
Maintenance	The upkeep, repair and/or routine and general cleaning of equipment and facilities
Microbiology	Activities involving the manipulation, isolation or analysis of microorganisms in their viable or infectious state
Molecular investigations	Activities involving the manipulation of genetic material from microorganisms or other infectious material for further analysis
Serology	Diagnostic examination and/or scientific study of immunological reactions and properties of blood serum
Hematology	Scientific study of the physiology of blood
Other	Other types of activity not captured elsewhere

**Table A2:** Definitions of occurrence types

Occurrence type	Definition
Spill	Any unintended release of an agent from its container
Loss of containment	Includes malfunction or misuse of containment devices or equipment and other types of failures that result in the agent being spilled outside of, or released from, containment
Sharps-related	Includes needle stick, cut with scalpel, blade or other sharps injury (i.e., broken glass)
Animal-related	Includes animal bites or scratches, as well as other exposure incidents resulting from animal behaviour (i.e., animal movement resulting in a needle stick)
Insect-related	Includes insect bites
PPE-related	Includes either inadequate PPE for the activity or failure of the PPE in some way
Equipment-related	Includes failure of equipment, incorrect equipment for the activity or misuse of equipment
Procedure-related	Includes instances when written procedures were not followed, were incorrect for the activity or were inadequate or absent

Abbreviation: PPE, personal protective equipment
